# Three Decades of Valproate: A Current Model for Studying Autism Spectrum Disorder

**DOI:** 10.2174/1570159X22666231003121513

**Published:** 2023-10-16

**Authors:** David Zarate-Lopez, Ana Laura Torres-Chávez, Alma Yadira Gálvez-Contreras, Oscar Gonzalez-Perez

**Affiliations:** 1 Laboratory of Neuroscience, School of Psychology, University of Colima, Colima 28040, México;; 2 Physiological Science Ph.D. Program, School of Medicine, University of Colima, Colima 28040, Mexico;; 3 Department of Neuroscience, Centro Universitario de Ciencias de la Salud, University of Guadalajara, Guadalajara 44340, México

**Keywords:** Autism spectrum disorder, valproic acid, HDAC inhibition, neurodevelopment, prenatal exposure, pathophysiology

## Abstract

Autism Spectrum Disorder (ASD) is a neurodevelopmental disorder with increased prevalence and incidence in recent decades. Its etiology remains largely unclear, but it seems to involve a strong genetic component and environmental factors that, in turn, induce epigenetic changes during embryonic and postnatal brain development. In recent decades, clinical studies have shown that in-utero exposure to valproic acid (VPA), a commonly prescribed antiepileptic drug, is an environmental factor associated with an increased risk of ASD. Subsequently, prenatal VPA exposure in rodents has been established as a reliable translational model to study the pathophysiology of ASD, which has helped demonstrate neurobiological changes in rodents, non-human primates, and brain organoids from human pluripotent stem cells. This evidence supports the notion that prenatal VPA exposure is a valid and current model to replicate an idiopathic ASD-like disorder in experimental animals. This review summarizes and describes the current features reported with this animal model of autism and the main neurobiological findings and correlates that help elucidate the pathophysiology of ASD. Finally, we discuss the general framework of the VPA model in comparison to other environmental and genetic ASD models.

## 1 INTRODUCTION

Autism Spectrum Disorder (ASD) is a complex neurodevelopmental disorder that affects communication, social interaction, and behavior [1]. In the past few decades, the incidence and prevalence of ASD have been increasing, making it a major public health concern worldwide [2, 3]. Despite extensive research, the etiology of ASD remains largely unknown, but it is widely accepted that genetic and environmental factors play a significant role in its pathogenesis [4, 5].

One such environmental factor that has been linked to ASD is in-utero exposure to valproic acid (VPA), a commonly prescribed antiepileptic drug [6], also used for the treatment of bipolar disorder, migraine, neuropathic pain, and headaches [7, 8]. Studies have shown that children exposed to VPA during pregnancy are more likely to develop ASD [9]. This association has been established through clinical studies where prenatal VPA exposure in rodents has been developed as a reliable translational model to study the pathophysiology of ASD [10]. However, the procedures used to replicate behavioral phenotypes have consistently differed in dosages, concentrations, the gestational development period of exposure to VPA, and the molecular and biological mechanisms proposed to explain the physiopathology associated with ASD. Several experimental models have been conducted on different species, including rodents, zebrafish, and non-human primates. Furthermore, recent advances have been made by using organoids from human induced pluripotent stem cells, which report gene expression patterns affected by VPA exposure and allow for correlation with the biological mechanisms proposed by animal studies [11-13]. Thus, increasing evidence suggests that VPA impacts the growth, migration, and differentiation of neurons and certain types of glia, as well as the development of functional synapses. This study aims to review the potential mechanisms by which prenatal exposure to VPA can result in changes to brain development in contrast to findings from other animal models and postmortem evidence from individuals diagnosed with ASD.

## 2 VALPROATE (VPA)

**Fig. (1):**
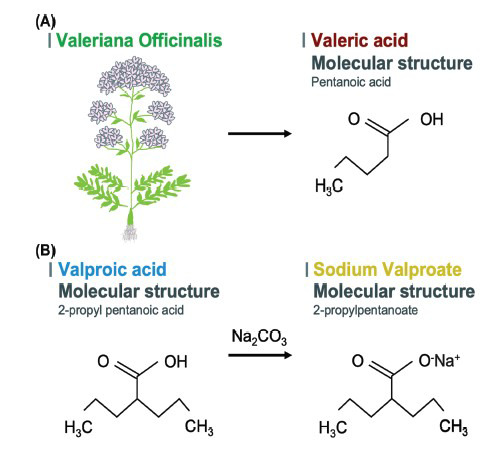
Molecular structure of Valproate (VPA). (**A**) Valproic acid can be chemically synthesized from valeric acid, a natural substance from *Valeriana officinalis.* (**B**) Valproic acid by addition of sodium hydroxide to obtain sodium valproate. Both are the most common forms of valproate in the clinical.

Valproic acid, also known as 2-propyl pentanoic acid, is a branched and short-chain fatty acid chemically produced by different synthetic pathways [14], one of them as a product derivate from valeric acid [8] (Fig. **1**). After oral administration, VPA is absorbed from the gut and metabolized in the liver by three routes, mainly glucuronidation, β oxidation in the mitochondria, and, in less proportion, by cytochrome P450-mediated oxidation [15, 16]. VPA has a high protein bound mainly to albumin and low clearance [15].

VPA was first introduced in the clinical as a broad-spectrum antiepileptic drug [17], which is currently used for the treatment of multiple seizure disorders [18], bipolar disorder as a mood stabilizer [19], migraine [20, 21], and schizophrenia [22]. VPA is also used for the treatment of pediatric diseases such as epilepsy, conduct disorder and for symptoms of irritability, aggression, and impulsivity [8]. VPA can cross through the placenta and accumulate in the fetal circulation with a higher concentration than maternal blood [23, 24], conferring a major risk to the fetus by exposure to VPA. On the other hand, VPA metabolites, such as 4-ene VPA, cannot cross through the placenta, and fewer concentrations are observed in comparison to VPA [24, 25].

## 3 STUDIES RELATED TO FETAL VPA SYNDROME AND ASD

Gestational exposure to VPA has been extensively associated with an increased risk of major congenital malformations, delayed cognitive development, and ASD [26-31]. Currently, its clinical recommendation for pregnant women is controversial [6, 32, 33].

Fetal valproate syndrome (FVS) was first reported in 1993 to describe a group of major congenital malformations associated with exposure to VPA during the first trimester [34]. Currently, there is a long list of congenital disturbances associated with FVS, including neural tube defects and skin, musculoskeletal, cardiovascular, genital, and pulmonary abnormalities [35-38]. Furthermore, fetal exposure to VPA is related to cognitive impairments and developmental delays [6, 39-44]. However, there is no clear linear relationship between VPA dose, exposure time window, and risk of major congenital or developmental delays arising from prenatal exposure to VPA. In general, the first trimester of pregnancy could be a period of susceptibility to the teratogenic effects of VPA during development [43-48]. However, this susceptibility may be explained by developmental processes during the first trimester of pregnancy. During this period, the processes of organogenesis and neural tube closure occur [49, 50], which are especially vulnerable to external factors such as infections, medications, and environmental toxins, which can interfere with the normal development of organs and structures and lead to congenital malformations [47, 51-53].

In addition, the first studies describing FVS also reported an increase in the incidence of autism diagnosis in children [27, 54-56]. Further studies support VPA during gestation as a risk factor for increased ASD diagnosis in children [28, 57-59]. In a longitudinal study with 528 children exploring the relationship between prenatal exposure to antiepileptic drugs and the prevalence of neurodevelopmental disorders, ASD was the most frequent diagnosis for the offspring of pregnant women taking monotherapy or polytherapy of VPA [60]. One of the largest studies, conducted from 1996 to 2006, with a sample of 655, 615 children, reported a two-fold increased risk of a diagnosis of ASD in children exposed to VPA in utero [31]. Further studies with large cohorts found similar results [39, 61, 62]. Curiously, fetal VPA exposure in any trimester of gestation had an increased risk of ASD diagnosis in children [9], which supports the importance of elucidating the mechanism of VPA in the cellular and molecular processes underlying ASD.

## 4 PRENATAL VPA EXPOSURE AS A MODEL OF ASD

According to clinical evidence, there is a link between maternal treatment with VPA and an increased risk of ASD diagnosis in their children. As a result, prenatal VPA exposure has been suggested as an experimental animal model for autism-like behaviors. The study of animal models that mimic some of the pathogenic mechanisms and clinical phenotypes observed in human disease can be valuable for developing new therapies that can be extrapolated to humans [63-65]. Currently, no medical test is available for diagnosing ASD, nor are there specific neurobiological markers associated with the disorder. Instead, clinicians rely on evidence from behavioral criteria described in DSM-V-TR [66], which limits the face validity criteria considered to model clinical phenotypes in animals. This also highlights the relevance of understanding the physiopathology of human disorders. Therefore, the face validity of prenatal VPA exposure is based on detecting behavioral impairments that resemble ASD in humans [67]. Also, this model partially achieves construct validity by using VPA, a widely described risk factor associated with ASD. The etiological and pathological mechanisms involved are not fully understood, but they are complex and heterogeneous conditions involving genetic and epigenetic interactions during early brain development. According to this, the prenatal VPA exposure model represents a form of idiopathic ASD that involves environmental factors.

Since the first study reporting prenatal exposure to VPA in rats as a model linked to autism [68], it has been extensively reported across multiple studies that reproduce the behavioral features associated with ASD. These features include lower sociability, deficits in communication, increased repetitive behavior and stereotypies, deficits in prepulse inhibition, lowered sensitivity to pain, increased anxiety, and hyperlocomotor activity [69-72]. Despite the promising results, there is considerable variability in the methods used to induce the VPA model. The first study reporting the critical method to induce autism-like behaviors in rats involved injecting pregnant rats with a single dose of VPA around the time of fetal neural tube closure, which occurs at approximately gestational day 12 [73]. One common source of variability is the gestational day of exposure to VPA. Studies choose to expose embryos earlier or later than day 12. Indeed, social deficits and sensory abnormalities have also been observed in rodents following early postnatal exposure to VPA [74-76]. This finding supports the notion that short gestational development in rodents is comparable to the early postnatal period in humans [77]. However, the effects of VPA on gene expression were different during the postnatal period [78]. Additionally, the dose of administration and sex-specific studies are common sources of variability between studies, especially in rats and mice, which are the most commonly used species for modelling purposes, as previously discussed [69, 79]. Despite the differences reported to induce an autism-like behavioral model, behavioral features and the pathophysiological mechanisms involved appear to be conserved across species. Several studies support the role of VPA in ASD in nonhuman primates, rats, mice, chicks, and fish (Table **1**). This makes it a valuable model for further research into the complex gene-environment interactions involved in ASD. In humans, maternal VPA is a risk factor because it affects fetal brain development but seems not to be associated with a specific trimester of gestation [9]. A systematic analysis conducted in rodents recapitulates the differences in dose and time of administration for the two major core symptoms of ASD. These symptoms are characterized by deficits in social interaction and communication, and an increase in restricted or repetitive/stereotyped behavior [66]. Moreover, there is an additional sub-category described as cognitive rigidity or inflexibility, concluding that despite methodological differences, a dose between 400-600 mg/kg and a time window around E11.5-12.5 consistently induce core behavioral impairments related to ASD. This holds true even across different rodent strains and routes of administration [80]. In addition, there is consistent evidence that a dose between 300-600 mg/kg administered around E10-E13 can induce autism-like behaviors.

Herein, we have compiled a summary of studies that have investigated the effects of prenatal exposure to VPA in rodents. We have focused on studies that used a consistent dosage range (400-600 mg) and administered the VPA around E11.5-E12.5 Additionally, we have included studies conducted in nonhuman primates, chicks, and fish that have reported behavioral impairments related to the two major core symptoms of ASD (Table **1**). In addition, the studies are classified according to the developmental period of the species during which the behavioral test was performed. In this sense, although the evaluation period has introduced additional variation into the model, the results have been conclusive.

## 5 VPA AND DISRUPTION OF BRAIN DEVELOPMENT

The mechanisms by which VPA disrupts development have not yet been fully explained. However, several reports have shown that prenatal exposure to VPA alters the processes of cell growth, neurogenesis, migration, differentiation, and apoptosis during gestational or early postnatal development [81, 82]. Multiple mechanisms have been proposed to explain how a single exposure to VPA in the period near the closure of the neural tube impacts the course of neural development and reproduces the morphological and functional changes found in ASD patients.

**Fig. (2):**
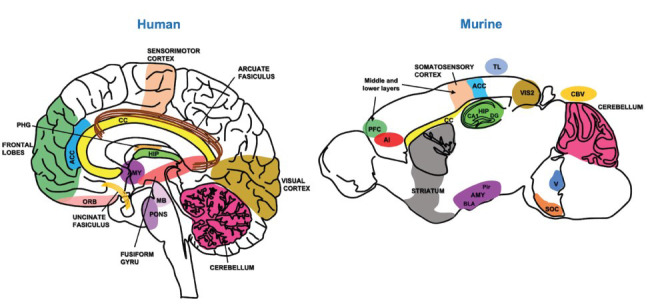
Anatomical and cellular disorganization in neuronal distribution and white matter circuits observed in the human brain of ASD subjects and the autism-like model of VPA exposure. **Abbreviations:** CC Corpus callosum, HIP Hippocampus, PHG Parahippocampal gyrus, AMY Amygdala, CBV Cerebellar vermis, ACC Anterior cingulate cortex, PFC Prefrontal cortex, V Motor nuclei, SOC Superior olivary complex, VIS2 Secondary visual area, DG Dentate gyrus, CA1 Hippocampal field CA1, AI Agranular insular area, TL Temporal lobe, Pir Piriform cortex, BLA Basolateral amygdala, MB Midbrain, ORB Orbitofrontal area.

Children with ASD commonly display a range of alterations of brain networks [199-201], which are considered a brain connectivity disorder that modifies excitatory/inhibitory balance [202]. However, several processes during brain development can affect this balance and the connectivity of several structures. Postmortem brain analysis of ASD patients reported alterations in cellular distribution, brain volume, and abnormal neuronal morphologies that affect their connectivity and functioning [82, 203-206]. In this section, we focus on how prenatal exposure to VPA in experimental animals replicates the clinical evidence reported in patients with ASD (Fig. **2**), which helps to understand the physiopathology of this disease.

### 5.1 Neurogenesis and Cell Growth

Neurogenesis is the process of generating new neurons by neural stem cells (NSCs) and neural progenitor cells (NPCs), which are regulated by epigenetic and genetic factors. Neurogenesis and cell growth are coordinated events during brain development and are essential to developing functional structures and neural circuits [207]. Therefore, neurodevelopmental disorders such as ASD are linked to alterations in this process.

It was proposed that abnormal growth patterns during brain development in the physiopathology of ASD [208, 209], which manifest as differences in head circumference or brain size [210-215], are even related to higher co-morbidity with macrocephaly and microcephaly [213, 216]. These macro anatomical changes could be related to the disruption of neurogenesis and cell migration during brain development. Postmortem brain tissue from young ASD patients has shown an increased neuronal population in the cortex [217-219], abnormal cortical lamination [219], and cerebral dysplasia in several regions of the brain [220]. However, evidence from brain postmortem or imaging in ASD patients is limited, hence the relevance of animal models.

Based on the evidence from ASD patients, several reports have indicated that prenatal exposure to VPA alters the processes of neurogenesis by affecting the population of NSCs and NPCs [185, 221, 222] that, in turn, reduces the number of neuroblasts and modifies their cell fate during embryo development [82, 223, 224]. Prenatal exposure to VPA in a window from embryonic day (E) 10.5 to E12.5 alters the neuronal distribution and density in the postnatal brain hippocampus, cerebellum, brainstem motor nuclei, superior olivary complex, and cerebellar vermis [129, 225-228]. Some studies even suggest differences in cortical layers affecting the neuronal distribution in the upper layers of the prefrontal, somatosensory, and secondary visual cortex [88, 229] and middle and lower layers of the prefrontal and somatosensory cortex [130, 230]. Consistent with this, hippocampal neurogenesis in the adult brain was found to be reduced after prenatal exposure to VPA [81], and cell density impairments were reported in the dentate gyrus and CA regions. Similarly, acute exposure to VPA during the entire gestational period inhibits the cell cycle exit of NPCs and increases the production of projecting neurons in superficial neocortical layers [231].

Interestingly, non-murine models of prenatal exposure to VPA, such as non-human primates and zebrafish, show a consistent neurogenesis impairment. Prenatal exposure to VPA in cynomolgus monkey at gestational day 26, which is an equivalent period in murine models, found a decreased neurogenesis and fewer cells in the cerebellar external granular layer and layers II and IV of the prefrontal cortex (PFC) [185]. Zebrafish embryos exposed to VPA after eight hours post fertilization showed an increased proliferation rate [193] and reported on this model delayed neurogenesis NeuroD1 disruption on the optic tectum, a brain region with homology to the superior colliculus in humans [232, 233]. In addition, studies *in vitro* have helped us understand VPA’s effects on neurogenesis and cell growth. Exposure to 1mM VPA decreases the proliferation of human NPCs in culture, followed by mitochondrial dysfunction and increased differentiation to an excitatory neuronal phenotype [234]. Interestingly, NPCs from rat embryos exposed to 0.5 mM VPA increase proliferation and differentiation to a neuronal phenotype [222], suggesting different effects according to the VPA concentration. Lastly, novel techniques such as three-dimensional cultures, called brain organoids, have become especially relevant for modeling brain development and neurodevelopmental disorders directly from human-derived cells [235, 236]. Single VPA exposure in brain organoids from human embryonic stem cells (hESCs) alters the neurogenesis and NPC population, which contributes to dysregulated neuronal fate toward upper-layer neurons, causing abnormal neocortical expansion [12]. The effects of the exposure to VPA appear to be time-dependent, as observed when a five-day exposure to 0.5 mM VPA in human-brain organoids shows an increase in the proliferation of neural precursors, but not after 10 days of VPA exposure [11]. This event seems to be dependent on Wingless (Wnt) signaling and Pax6 transcription factor expression [122, 222] that, in turn, can lead to differential outcomes in the total number of mature neurons. Overall, these results suggest a dysregulation in the early processes of neurogenesis and cell growth arising from prenatal exposure to VPA, similar to what occurs during neurodevelopment in patients with ASD.

### 5.2 Migration

Neural migration is a crucial event in the development of brain circuits (Pan *et al.*, 2019). In this process, nascent neurons undergo cellular migration from the ventricular-sub-ventricular zone [237] in a programmed pattern to align radially into columnar structures, providing a functional unit in the neocortex [224, 238]. Disruption in the number of newborn cells or the migration process could lead to the abnormal neuronal distribution discussed in the previous section. Interestingly, abnormalities in cortical lamination have been previously reported in ASD patients [239-242]. Thus, prenatal VPA exposure disrupts granule cell number during the migratory period of cerebellar development, causing abnormal Purkinje cell layer [243], and exotopic Purkinje cells have been identified [82]. A study comparing migration patterns of BrdU+ cells from mice embryos exposed to VPA on days 12.5, 13.5, or 14.5 found less number of cells incorporated in cortical layers on postnatal day 7 by prenatal exposure on day E12.5 Interestingly, prenatal exposure on day 13.5 slightly reduces the number of BrdU+ cells, and no differences were reported in migration patterns by prenatal exposure on day 14.5 [130]. Previous studies reported that prenatal exposure to VPA alters the migration of NPCs in the adult hippocampal neurogenesis and decreases the expression of Cxcr4 [244], a chemokine related to proper migratory patterns of granule newborn neurons in neurogenesis [245, 246], suggesting a mechanism implicated in seizure susceptibility [244]. These disturbances of the migration pattern of new neurons in the adult hippocampus caused by prenatal VPA exposure are related to increased seizure susceptibility [244]. However, changes in CXCR4 transcription have been directly linked to the inhibition of histone deacetylase activity (HDACi) by VPA, thus increasing Histone H3 acetylation at the promoter site for *Cxcr4* in cultured mesenchymal cells, increasing their ability to migrate [247]. In addition, several genes involved in cell adhesion and migration processes are differentially expressed after prenatal VPA exposure [244], including *Cntnap2*, a gene recently associated with ASD [248]. Therefore, transcriptome dysregulation of genes regulating cell migration could be a mechanism related to the pathophysiology of ASD.

### 5.3 Cellular and Molecular Impairments in Neuronal Organization

During brain development, neurons exhibit unique morphological and molecular changes that give rise to broad neuronal phenotypes and complex neural circuits. These structural and functional changes include a long axonal process to connect with target neurons, complex dendritic arborization, the establishment of functional synapses, specific molecular profiles such as neurotransmitters, and properties of neural firing reflecting functional phenotypes [249, 250]. Furthermore, the neuronal arrangements lead to complex connectivity and brain function [251]; therefore, disrupting these processes could lead to dysfunctional networks [224]. In addition, ASD was previously suggested as a heterogeneous group of disorders emphasizing differences in brain growth and molecular phenotypes [208].

Postmortem brain analysis of ASD patients showed an increased number of 5-HT+ ascending fibers from serotonergic neurons of the medial/lateral forebrain bundles, including increased innervation density into the amygdala, piriform, superior temporal, and parahippocampal cortices [252, 253]. Furthermore, small soma in neurons and increased cell density per area were reported in the hippocampus, cerebellum, and frontal and temporal lobes of ASD patients [206, 254]. In addition, decreased density of dendritic spines was reported in the prefrontal cortex and hippocampal neurons [255-258]. According to these findings, prenatal VPA exposure generates large soma in neurons from cerebellar nuclei [227] and altered dendritic arborization patterns by higher complexity in the proximal dendritic segment, but fewer branches at distal sites of cerebellar Purkinje cells [228]. Besides, decreased dendritic density or spine density has been reported in the hippocampus, amygdala, prefrontal and somatosensory cortex [81, 82, 259, 260]. Previous studies have reported abnormal neuron compartmentation of cells in the striatum, which leads to decreased corticostriatal synapses and impaired circuits with the prefrontal, granular insular, and somatosensory cortices because of prenatal VPA exposure [88].

In addition, studies *in vitro* and on zebrafish have been helpful in understanding the mechanisms implicated in cellular and molecular impairments arising from prenatal exposure to VPA. Axonal ectopic branches and excessive abnormal branching increased in a VPA concentration-dependent pattern during the gestational development of zebrafish [261]. In contrast, at higher doses, VPA induces delayed neuropil formation and axogenesis [232]. Interestingly, VPA induces abnormal differentiation of serotoninergic neurons by downregulating the proneural ascl1b gene, a mechanism mediated by HDAC1 inhibition [262]. The effect on neuronal differentiation could be dependent on the period of development. In culture neurons from mice, embryos exposed to VPA at day 12.5 but not E14.5 had reduced dendritic morphology and expression of synaptic proteins such as *Nlgn1*, *Cntnap2*, and *Shank3*, all of which are associated with ASD [263].

### 5.4 Excitatory/Inhibitory Imbalance

The excitatory and inhibitory (E/I) neural activities are highly regulated at molecular, cellular, and circuitry levels in the nervous system. These changes maintain a relatively stable relationship through multidimensional and time-scaled neural circuits in the brain. Otherwise, structural alterations, especially during the brain development of cortical layers and synapse formation, could lead to dysregulated synaptic transmission, plasticity, and intrinsic neuronal excitability. These factors may contribute to an imbalance in E/I signaling, which could explain the etiology of ASD [202, 264]. The term was first introduced to explain sensory, social, and emotional behaviors in individuals with ASD [265]. Binocular rivalry is a perceptual process that emphasizes the importance of reciprocal inhibition [266, 267], which is dependent on E/I dynamics and is reduced in individuals with ASD [268, 269]. The distribution of neurons in the neocortex is arranged in mini-columns, which modulates the microcircuitry of glutamatergic and GABAergic signaling. This arrangement is altered in ASD subjects, leading to impaired integration [254, 270-272] and processing of signals [273]. In addition, lower GABA concentrations in the sensorimotor cortex correlate positively with increased sensitivity to tactile stimuli in ASD adult subjects [274]. However, differences in neurotransmitter concentrations may not be the only factor that explains these differences in sensory processing. A study found no variations in GABA levels in adults with ASD of similar age [275]. In addition, human postmortem studies have reported decreased levels of GABA_A_ and GABA_B_ receptors in the anterior cingulate cortex [276, 277] and dysregulated expression of glutamate decarboxylase enzymes *GAD65* and *GAD67* in the cerebellum [278-280]. Also, several genetic variants in GABA receptor subunits have been reported in individuals with ASD [281]. Both increased and decreased ratios of E/I activity have been suggested in ASD subjects. This could be attributed to various compensatory mechanisms that underlie the heterogeneity of the ASD population [282].

Prenatal exposure to VPA induces a decrease in the excitability of glutamatergic neurons and an increase in local connectivity of the prefrontal cortex in adult rats but not in adolescence [283]. This could be explained as an impaired compensatory mechanism or the result of delayed prefrontal cortex maturation. In addition, altered expression of glutamatergic (vGluT1, GluN1-2, PSD-95) and GABAergic (vGAT, GAD65, GAD67) proteins, as well as changes in the number of synapses, were reported in adolescent and adult mice exposed to VPA [284]. The same increased imbalance in E/I proteins was reported in the prefrontal cortex [95, 285]. Additionally, pharmacological blockage of the glutamatergic transmission reverses the impairments in social behavior [285]. Selectively inhibiting D2R+ pyramidal neurons in the medial PFC ameliorates social deficits, whereas non-specific inhibition does not improve social behavior [286]. In the somatosensory cortex, an overexpression of NMDA receptor subunits and enhanced mediated transmission and LTP were reported in rats prenatally exposed to VPA [287]. Moreover, local hyperconnectivity and reduced excitability of pyramidal neurons have been reported in this region, specifically the PFC [288, 289], which contribute to the altered microcircuitry of glutamatergic and GABAergic signaling. In addition to this E/I imbalance, the number of PV+ GABAergic interneurons was reduced in the neocortex [229], and inhibitory post-synaptic currents were reduced in the temporal cortex [290]. Consistently, studies have reported abnormal amygdala synaptic E/I imbalance and hyper-excitability [131]. In the anterior cingulate cortex, there was a significant decrease in the E/I imbalance during the postnatal development of mice from P7 to P30. This imbalance resulted in altered transmission and synaptic plasticity, which were associated with decreased BDNF expression during the developmental period [291]. Abnormal development of synaptic transmission was also reported in the cerebellum of VPA-treated mice [228]. In addition, nonhuman primates exposed to VPA during gestation showed impaired levels of proteins related to glutamatergic (vGluT2, mGluR5, GluN2) and GABAergic (vGAT) systems [185]. Also, a study reported a differential effect on expressed genes in E/I neurons in human forebrain-derived organoids exposed to VPA [13]. Overall, these results support the hypothesis that prenatal exposure to VPA exposure leads to an E/I imbalance, which is associated with the physiopathology of ASD.

### 5.5 Oligodendroglia and Myelination Impairments

Oligodendrocytes (OLs) and myelination play critical roles in shaping the functional and structural connectivity of the central nervous system. OLs are glial cells responsible for the production and maintenance of myelin sheaths around axons. They facilitate neuronal transmission and provide trophic and metabolic factors [292]. Oligodendrocyte precursor cells (OPCs) have been identified since E12-E14 in rodents and gestational weeks 6-10 in humans. However, myelination begins late in gestational development in humans and postnatally in rodents [293-295].

Abnormalities in the development of myelination and white matter integrity have been reported in ASD subjects, which disrupt the connectivity of inter-hemispheric, short-local, and long-range association fibers, resulting in abnormal brain growth patterns [296-299]. Enlargement of white matter structures during the first years of life is described in individuals with ASD, but it tends to decrease in size as they age [300]. A study conducted with ASD adolescents revealed a low density of white matter tracts in the corpus callosum and long-range association fibers, such as the uncinate and arcuate fasciculus [301]. Also, a combination of decreased and increased white matter across brain structures has been reported, emphasizing age-related differences [302]. Thus, pathological changes in the cytoarchitecture of the white matter in the cerebral cortex and abnormal myelination in the corpus callosum may explain the dysfunctional connectivity found in several regions of ASD subjects. In addition, the presence of white matter abnormalities or their resolution is associated with the severity of symptoms [303-310]. Abnormalities in the population of OLs and axon myelination could explain these differences.

Prenatal exposure to VPA resulted in a decrease in myelin basic protein (MBP) immunoreactivity in the basolateral amygdala and piriform cortex of adult mice, which correlates with impaired social behavior. Additionally, the basolateral amygdala and piriform cortex exhibited an increase in myelin sheath thickness and a decrease in the number of Olig2+ and CC1+ cells. Olig2 is a transcription factor for the OL lineage, while CC1 is a common marker of mature OLs. These changes were observed in both the PFC and piriform cortex [299]. However, differences in age, brain structures, and the process of oligodendroglia maturation have been reported. In rats prenatally exposed to VPA, variations in *Olig2* messenger RNA (mRNA) and protein levels, as well as the number of Olig2+ cells, were observed in the hippocampus, PFC, and cerebellum [83, 311]. Also, differences were reported between early, juvenile, and adult development, with both increased and decreased patterns of Olig2 marker or positive cell numbers [311]. In contrast, a study reported a decrease in myelin content but a preserved population of OL cells in the corpus callosum at PND15. However, there was a consistent disruption observed in myelin content, number of myelinated axons, and OL population from PND15 to PND36 [312]. These stages in rats are similar to the infant and juvenile stages of human development [197]. Overall, these results suggest a significant reduction in the postnatal development of OLs and myelination in the corpus callosum. Additionally, prenatal exposure to VPA appears to affect the trajectories of OL and myelination in the gray matter differently. According to this, there is a consistent finding of reduced white matter density and structural integrity of the corpus callosum in ASD subjects, regardless of age. However, there are different patterns observed in gray matter structures and long-range association fibers [302]. In addition, downregulated expression was reported in *Olig2*, *Mbp*, and *Chd7*, while upregulated expression of *Lingo1* was observed in the PFC of prenatally exposed rats [313]. In this sense, CHD7 is a chromodomain helicase DNA-binding protein that promotes OL differentiation [314], and LINGO1 is a transmembrane signaling protein that inhibits OL differentiation and myelin production [315]. Overall, a few studies have addressed the impairments in OLs and myelination caused by prenatal exposure to VPA. Nevertheless, further studies are needed to clarify the mechanisms involved during gestational development and their relationship with ASD.

### 5.6 Immune and Oxidative Stress Impairments

Prenatal insults, such as infections, toxins, and maternal stress, as well as subsequent immunological activation, can increase the risk of neurodevelopmental disorders [316]. The mechanism by which maternal immune activation (MIA) leads to ASD has not been fully understood. This includes several pathological processes, such as the upregulation of cytokine and chemokine expression, oxidative stress, mitochondrial dysfunction, early glial activation, and maternal autoantibodies that cross to the fetus [317-321]. These processes result in long-lasting changes in the expression of other immune molecules, such as major histocompatibility complex I and molecules that regulate synapse formation and brain development [317].

MIA plays a significant role in the heterogeneous and biological etiology of ASD [322]. In individuals with ASD, decreased mononuclear cells, T lymphocytes CD4+, low responsiveness to mitogen stimulation [323, 324], and increased levels of pro-inflammatory cytokines [325-327] have been reported. Postmortem studies have reported increased activated microglia and reactive astrocytes in the cerebellum, fronto-insular cortex, prefrontal cortex, and visual cortex [328-330]. Noninvasive neuroimaging studies with (^11^C)(R)-PK11195, a selective radioligand for microglia, have shown an increased activated phenotype in several brain regions, including the cerebellum, midbrain, pons, fusiform gyri, anterior cingulate, and orbitofrontal cortex [331]. In addition, MIA involves oxidative stress and mitochondrial dysfunction [332, 333]. Moreover, in individuals with ASD, impaired production of oxidative markers such as reactive oxygen species (ROS) and nitrogen free radicals [317, 318, 334] has been reported. Additionally, decreased levels of glutathione (GSH), oxidized glutathione (GSSG), and glutathione redox/antioxidant capacity (GSH/GSSG) [318] suggest increased oxidative stress and reduced antioxidant capacity.

Although VPA exposure is not an infectious factor, prenatal exposure has been shown to induce immune activation in the brain. In murine models, an increased density of astrocytes and microglia was reported in the prefrontal cortex, hippocampus, and cerebellum [154, 311, 335]. In the hippocampus, prenatal exposure to VPA increased the expression of anti-inflammatory microglial M2 phenotype markers. Otherwise, in the cerebral cortex, both M2 and pro-inflammatory M1 phenotype markers are increased, which is consistent with the increased expression of pro-inflammatory cytokines only in the cerebral cortex [336]. An increase in ROS and a limited antioxidant capacity have also been demonstrated in both regions [336]. This could potentially trigger immune activation. Also, TREM2 downregulation, a transmembrane immune receptor expressed exclusively in microglial cells, has been proposed as a mechanism related to the activation of a pro-inflammatory phenotype and its role in synaptic pruning [337]. In contrast, a reduction in Iba1+ microglia was observed in the motor cortex, which may be attributed to the early postnatal age [338]. In addition, studies have reported increased responses to inflammatory stimuli and elevated basal levels of corticosterone [149, 154, 180], which have both suppressive and enhancing effects on immune function [339]. According to this, prenatal exposure to VPA induces atrophy of the thymus [135, 180]. It also leads to lower levels of IFN-γ/IL10 and increased production of nitric oxide (NO) in peritoneal macrophages [180]. In fact, the levels of both IFN-γ and NO were positively correlated in individuals with ASD [340]. In contrast, several studies have reported the anti-inflammatory properties of histone deacetylase (HDAC) inhibition with VPA [341-343]. However, increased pro-inflammatory cytokines TNFα, NO, and IL-1β were reported after acute exposure to VPA in macrophages, but only in response to an inflammatory stimulus [344]. Blood-brain barrier (BBB) impairment during the gestational period was also suggested as a pivotal event to increase immune activation [345]. Accordingly, prenatal exposure to VPA causes impaired BBB permeability and aquaporin expression in the choroid plexus, prefrontal cortex, and somatosensory cortex [346]. Overall, this evidence supports immune alterations caused by prenatal exposure to VPA during postnatal brain development or in adult mice.

## 6 VPA MECHANISM OF ACTION DURING GESTATION

The long-term behavioral and neurobiological impairments associated with ASD in human patients caused by VPA are not completely elucidated in terms of how they begin after a single VPA exposure. However, we summarize these neurobiological alterations in the postnatal brain in Table **2**. Several studies have suggested a set of intersecting pathways and multiple chemical interactions with VPA [69]. Some of these interactions have been experimentally demonstrated, such as HDAC [347, 348], while others have been suggested theoretically or *in silico*, such as GSK3β, PKCβII, JARID1A, and EZH2 [349-351]. Furthermore, it is not clearly understood how HDAC inhibition leads to several dysregulated processes during gestational development, which we will discuss in the following section. Additionally, it is unclear how these disturbances converge with other ASD-related animal models, suggesting a complex genetic-epigenetic interplay associated with the etiology of ASD [352].

### 6.1 Epigenetics: HDAC Inhibition and Chromatin Remodeling

VPA can regulate gene expression due to its mechanism as a histone deacetylase inhibitor (HDACi). HDAC is an enzyme responsible for removing acetyl groups from histone proteins, which can result in the tightening of chromatin structure and the repression of gene expression [353]. Conversely, HDACi, such as VPA, increase the level of histone hyperacetylation associated with a more open chromatin structure, allowing for increased accessibility of DNA to transcription factors and other regulatory proteins [353].

VPA and its analogs inhibit multiple HDACs from Classes I and II (excluding Class IIb, which is composed of HDAC6 and 10). This inhibition leads to an increase in histone H3 and H4 acetylation [347, 348, 354], specifically at lysine (K) residues [355]. The acetylation levels of H3/H4 were transiently increased after embryonic exposure to VPA in mice, which also exhibited autism-like behaviors [130]. H3K9ac was increased after VPA exposure in mouse embryonic stem cells starting on day 14 [356]. This is a critical histone modification that helps regulate embryonic stem cell pluripotency and neural differentiation [357, 358]. In addition, H3K9 is deacetylated by HDACs from Class I [359], which are highly expressed during mid-late embryonic development [360]. Additionally, the hyperacetylation pattern of Histones induced by VPA HDACi resulted in an increase in gene expression at promoter sites, including the *CDKN1A* promoter region (p21^Cip/WAF1^). In this sense, VPA increases hematopoietic cell differentiation in a p21-dependent manner through increased HDAC inhibition [347]. Interestingly, deficient HDAC1 activity during mid-late embryonic development was directly related to the up-regulation of p21 [360]. Also, lysine acetylation at the core histone domain, such as H3K56, was increased by VPA exposure [371]. H3K56 is located at the entry-exit sites of the DNA wrapped around the nucleosome. Acetylation of these sites modulates the unfolding of nucleosome-chromatin [372, 373], and it has been previously suggested that this process of chromatin remodeling relocates developmental genes, allowing the recruitment of transcription factors to promote cellular differentiation while downregulating genes that maintain pluripotency [374]. Mouse ESCs exposed to VPA undergo a significant change in chromatin accessibility. Specifically, there is a shift from pluripotency factors such as *Pou5f1*, *Nanog*, and *Sox2* to specific loci associated with chromatin remodeling and neuronal differentiation. One of the loci affected by this switch is *Pax6* [371].

In addition to the relationship between HDAC inhibition by VPA and ASD, several compounds analogous to VPA in chemical structure, such as valpromide (VPD), which lack the effect of HDAC inhibition, did not induce autism-like behaviors in murine models or cause abnormalities in brain development [130]. Also, the epigenetic effect of VPA on other pathways was not observed with non-HDACi analogs. The inhibition of NPC proliferation mediated by Wnt signaling in brain organoids exposed to VPA was not replicated with VPD [12].

Consequently, prenatal exposure to VPA induces a sequential chain of events, starting with HDAC inhibition and leading to changes in developmental transcriptional profiles. As a result, this leads to increased activity of transcription factors and, indirectly, abnormal neural proliferation and differentiation that may arise from these early epigenetic modifications. Chromatin immunoprecipitation demonstrated increased binding of acetylated histones to the *Pax6* promoter region, which leads to transient up-regulation of Pax6 expression and increased glutamatergic differentiation in the prefrontal cortex in VPA-treated embryos [122]. The same results were reported in mouse ESCs exposed to VPA, which led to an increase in H3K56ac locus-specific gain of function within the *Pax6* promoter [371]. This transcription factor regulates the balance between neural stem cell (NSC) proliferation and their differentiation into neurons [375, 376]. It specifically affects the development of glutamatergic phenotypes derived from the ventricular zone of the dorsal telencephalon that migrates into the cortex [377]. Interestingly, the outcome of neural differentiation could vary depending on the timing and level of Pax6 expression. This is due to the gain-of-function or loss-of-function effects caused by the transcriptional regulation of Pax6 on self-renewal, neurogenesis, and the cohort of genes that determine cell fate [378]. Also, a predicted increase in occupancy of the *Gabpa* transcription factor was reported after VPA exposure in mouse ESCs [371], which binds to the *Tert* promoter to enhance its expression [379]. In addition, VPA exposure increased histone acetylation at the *Tert* promoter region, as demonstrated *in vitro* in NPCs and *in vivo* by E14 embryo brains from rats prenatally exposed to VPA at E12 [361]. This increase also led to enhanced Pax6 and Brg1 immunoprecipitation, which subsequently recruited transcription factors that determined glutamatergic neuronal differentiation. In contrast, H3K56ac locus-specific loss of function within *Asf1a* was reported after VPA exposure in mouse ESCs [371]. The downregulation of their expression was associated with a decrease in pluripotency markers (Nanog, Sox2, Oct4) and an increase in differentiation markers (Sox17, FoxA2, Pax6) [380]. Consistently, VPA and MS-275, both HDAC inhibitors, increased the expression of the pre-synaptic glutamatergic vGluT1 vesicle transporter and decreased the expression of GABAergic markers such as vGAT, GAD65, and GAD67 in cultured cortical neurons [284]. In contrast to this gain-of-function in glutamatergic neuronal differentiation, *Chd7* binds strongly to H3K27ac and *Sox10*/*Olig2* chromatin enhancers in OPCs to promote oligodendroglial lineage differentiation [314]. However, *Chd7* expression is downregulated in the PFC of rats exposed to VPA prenatally [313]. Although these differences were reported in the postnatal brain, OPCs have been identified as early as E12 in mice, which is a common timeframe for VPA prenatal exposure. In a similar way to how VPA inhibits HDAC function, a mouse model of HDAC1 or HDAC2 loss-of-function by conditional knockout (cKO) promotes β-catenin translocation into the nucleus and its stabilization with transcription factors to repress OL differentiation [381]. In addition, Chd7 loss-of-function decreased GABAergic differentiation during embryonic development [382], which can also contribute to an imbalance between E/I signaling in ASD.

Although VPA is a well-recognized HDAC inhibitor, the complex epigenetic interactions resulting from the hyperacetylation state are not fully elucidated. In this sense, HDACi allows for an open chromatin state while also facilitating access to other modulatory enzymes. Previously, it was suggested that the longer open state of chromatin observed after VPA treatment could be attributed to DNA and histone methylation [383]. Prenatal exposure to VPA increases the demethylation and expression of Wnt1 and Wnt2 ligands. It also upregulates mRNA levels of the downstream target genes *En1* and *Ccnd1* in the prefrontal cortex and hippocampus [363]. In addition, VPA increases both DNA demethylation of the *Reln* and *Gad67* promoters and acetylated H3 binding to the promoter regions of these genes [384, 385]. This effect of VPA was previously suggested to depend on DNA demethylase activity [386]. Accordingly, a passive mechanism was proposed that involves the decreased expression of DNA methyltransferase 1 (DNMT1) [387-389]. Additionally, an active mechanism was identified involving the activation of a DNA demethylase [390, 391], which acts on methylated CpG sites in gene promoters [392]. However, multiple mechanisms could interact in a dependent manner with histone acetylation changes. Previously, it was reported that VPA increases the expression of fat mass and obesity-associated protein (FTO). This protein suppresses the posttranscriptional processing of *Mbd2* mRNA, thereby affecting its function as a CpG demethylase over the *Scn3a* promoter region [393]. These results in indirect downregulated expression by methylation of *Scn3a* are evoked by HDAC inhibition.

Class I HDACs are contained in multiprotein complexes that commonly repress transcription [394]. Previously, it was demonstrated that VPA inhibits HDAC, thereby relieving transcriptional co-repression of PPARδ. According to this, VPA does not directly increase the expression of the PPARδ transcription factor. Instead, it downregulates the corepressor complex, which allows PPARδ to bind co-activators and enhance its transcriptional activity [395]. In addition, the interaction between PPARδ and VPA was ruled out. Therefore, it is more likely that HDAC3 inhibition, which is a core component of the nuclear corepressor complex, is responsible. Instead, acetyl groups are preserved, which partially facilitates the recruitment of co-activators [396]. According to this, VPA has the lowest IC_50_ for class I HDACs (HDAC 1-3) [347].

These corepressor complexes also include specific histone demethylases (HDMs), which are commonly involved in the combination of histone modifications [397]. According to this, both histone lysine methylation and demethylation were reported after exposure to VPA [398]. In contrast to histone acetylation, which promotes gene expression, methylation can either stimulate or suppress gene expression, depending on specific residues. In this regard, histone 3 at lysine 4 (H3K4) dimethyl and trimethylation (H3K4me2/me3) promote gene expression, and lysine 9 methylation (H3k9me) promotes gene repression [394]. Both H3K4 histone modifications were increased after VPA exposure in mouse embryonic stem cells [366], in rats exposed prenatally by E9 [362], and in cultured astrocytes and postnatal cortical neurons [399]. In contrast, prenatal exposure in rats decreased H3K9 monomethylation. However, the mechanism by which VPA induces both increased and decreased histone methylation is not fully understood. Previous studies suggest that crosstalk between histone modifications is facilitated by a complex that induces both regulatory enzymes. This crosstalk involves methylase activity-dependent substrate acetylation as well as the direct effects of VPA on EZH2 methyltransferase and JARID1A demethylase [350, 387, 400]. Moreover, histone methylation is more stable and can last for several days [383]. Increased H3K4me2 persisted for five days after exposure to VPA in cultured cortical neurons [399].

Lastly, in addition to the complex epigenetic effects of VPA, there has been a recent review of the emerging role of histone modifications and HDAC enzymes in the alternative splicing transcription of mRNAs. In general, chromatin remodeling not only enables the recruitment of regulatory enzymes and transcription factors but also can influence the timing of spliceosome complex coupling to exonic and intronic splicing sites of DNA, as well as the recruitment of chromatin-splicing adaptor proteins [401]. According to this, HDACis such as Trichostatin A (TSA) or VPA can promote H4 acetylation around splicing sites instead of promoter regions, which affects the sequential events and leads to an increase in alternative splicing of mRNAs [402]. Moreover, differentiated excitatory neurons from hiPSCs treated for 24 hours with VPA showed an increase in splicing transcriptional profiles from several genes related to ASD, as well as chromatin and transcriptional regulatory genes [365]. Given the significant importance of spatiotemporal expression patterns of alternative splicing during brain development [403] and the growing evidence of abnormal splicing variants in ASD [404], future studies should prioritize the identification of this emerging *in vitro* evidence from splice variants in brain development following prenatal exposure to VPA.

Overall, the mechanism of the VPA by HDACi could be strongly related to the heterogeneity of ASD, which is associated with genetic changes. Recently, a study was conducted using 45 postmortem brain samples from ASD subjects. The study demonstrated a shared histone-acetylome pattern in 68% of individuals, including both syndromic and idiopathic forms of ASD [405]. Interestingly, common pathways were associated with these epigenetic modifications, such as synaptic transmission, histone deacetylation, and immunity. The genes associated with ASD primarily encode synaptic proteins, transcriptional regulators, and chromatin remodeling factors, suggesting that synapse formation and the establishment of neuronal circuits during brain development play a crucial role in ASD [367].

### 6.2 Signaling Pathways

Previous studies suggest several signaling processes as mechanisms of action of VPA, including Wnt/β-catenin, PI3K/Akt, and MAPK/ERK pathways [69, 349, 406]. However, it is still unclear how many of these processes are dysregulated as a direct result of a VPA mechanism of action. Otherwise, it has even been suggested that dysregulation of Wnt/β-catenin may be a downstream event in the mechanism of action of VPA on HDAC [369]. Therefore, this section focuses on the direct evidence from VPA as a mechanism of action during gestation on signaling processes and their role in cellular processes that may alter neuronal and network development related to ASD etiology.

The Wnt signaling pathway plays a key role in embryonic development, regulating processes such as cellular growth and proliferation, migration, maintenance of stem cells, and neuronal polarity [407, 408]. The canonical Wnt signaling pathway leads to increased levels of β-catenin, which translocate to the nucleus, promoting the transcription of Wnt-dependent genes. β-catenin is regulated by the phosphorylation of glycogen synthase kinase-3β (GSK-3β), which leads to its degradation and the stabilization of cytoplasmic β-catenin [349, 409]. Some studies suggest that VPA stimulates the canonical Wnt signaling pathway through the modulation of HDAC and GSK-3β [406, 409]. VPA is proposed to activate Wnt-dependent gene expression through its HDACi activity [349, 410-412]. Prenatal exposure to VPA leads to the upregulation of the Wnt/β-catenin signaling pathway through increased degradation of GSK-3β and subsequent elevation of β-catenin levels [342, 363]. Also, VPA upregulates the Wnt/β-catenin pathway by increasing the phosphorylation of GSK-3β and decreasing the phosphorylation of β-catenin. This leads to a decrease in the expression of the redox proteins Trx1 and Trx2 and an increase in the expression of the oxidative stress marker 4-HNE in the prefrontal cortex and hippocampus [174]. Wnt ligands are glycoproteins secreted in an autocrine or paracrine fashion, interacting with the Wnt inhibitory factor and the Fzd receptor to transmit the signal downstream [413]. Prenatal exposure to VPA increases the expression of Wnt1 and Wnt2 ligands and upregulates mRNA levels of the downstream target genes *En1* and *Ccnd1* in the prefrontal cortex and hippocampus [363].

In addition, altered signaling of the Wnt/β-catenin pathway has been reported in ASD [409, 414], including variants of Wnt ligands [415-419]. Also, the chromodomain helicase DNA binding protein 8 (CDH8) has been identified as a significant candidate gene for ASD [420, 421]. One of the pathways regulated by CHD8 is the Wnt signaling pathway [422]. The Phosphatase and tensin homolog (PTEN) is another major candidate gene for ASD that interacts with the Wnt pathway [423]. This signaling protein is known to be an important regulator of neural circuit formation [408, 424], indicating that these pathways play a role in neural proliferation, migration, and differentiation during embryonic brain development. It is possible that Wnt/β-catenin signaling is dysregulated in ASD [406, 425].

Overall, this evidence suggests a strong modulation of Wnt signaling following exposure to VPA. Although it has been previously suggested that there is crosstalk between multiple signaling processes, few studies have been conducted during the gestational period or under similar *in vitro* conditions to explain these interactions. In this sense, the effect of VPA on β-catenin stabilizes Ras, promoting ERK-p21^Cip/WAF1^ signaling. This signaling pathway then promotes the differentiation and inhibits the proliferation of embryonic E14 NPCs [369]. Lastly, mTOR signaling through PI3K/Akt or PTEN modulation can contribute to the differentiation-induced process by VPA [426-428].

### 6.3 Oxidative Stress and Immune Response

The epigenetic effects and downstream signaling pathways altered by VPA exposure can partially explain an early oxidative stress environment contributing to impairments during embryogenesis and immune activation. Previously, an upregulated transcription of immune system pathways was reported to change across postnatal amygdala development in dams prenatally exposed to VPA [429]. In addition, exposure to gestational VPA before neural tube closure (E9) in mice increases ROS production and the occurrence of neural tube defects [430]. In contrast, the postnatal brain of prenatally exposed VPA showed a continued decrease in the expression of redox proteins and an increase in the expression of oxidative markers and pro-inflammatory cytokines [174, 336]. Although some of these changes could be partly explained by the epigenetic effect of VPA on transcriptional profiles, as previously suggested [429], further studies must elucidate their role through direct or indirect modulation, such as Wnt/β-catenin [174].

## 7 CONVERGENCE OF PRENATAL VPA EXPOSURE COMPARED TO OTHER MODELS OF AUTISM-LIKE BEHAVIOR: IDIOPATHIC AND SYNDROMIC FORMS OF ASD

ASD is frequently described as syndromic when its etiology is related to a single gene mutation or chromosome abnormality that explains a syndrome with a high prevalence of ASD diagnosis. Examples of such syndromes include Phelan-McDermid’s syndrome, which is often associated with a *SHANK3* mutation; Rett’s syndrome and MecP2 duplication syndrome, which are associated with *MECP2* mutations; Fragile-X syndrome, which is associated with *FMR1* mutations, Tuberous sclerosis complex, which is associated with *TSC1/2* mutations, Angelman’s syndrome, which is associated with *UBE3A* mutations, Prader-Willi’s syndrome, which is associated with chromosome 15q11-q13 deletion, and *CACNA1C* mutations [431]. On the other hand, non-syndromic ASD cases, which do not have identified etiological factors, are often referred to as idiopathic [432]. Both concepts are frequently applied to animal models, including prenatal exposure to VPA as an idiopathic model.

Also, risk factors associated with the incidence of ASD can be classified into environmental factors, epigenetic changes, and genetic variants [433]. Nevertheless, boundaries between this classification can be diffuse, given that environmental factors can cause epigenetic modifications. However, it is important to note that not all of the epigenome results from an environmental insult. It also depends on the interaction with specific genetic patterns and the predisposition of genetic variants [434]. Moreover, ASD-related genetic variants could lead to epigenetic modifications, such as MecP2 variants [435]. Instead, the etiology of ASD could be better conceptualized as an interplay of genetic-epigenetic interactions that affect specific pathways in brain development. This supports the observed complex heterogeneity in ASD [436]. According to this view, environmental risk factors such as VPA primarily increase the epigenetic load for ASD. On the other hand, single, well-identified mutations mainly increase the genetic load. Each case contributes to compromising shared pathways during brain development. Interestingly, a novel histone methyltransferase (EHMT1) loss-of-function syndrome in mice results in reduced levels of H3K9me3 in the embryonic brain at E11.5 This syndrome also leads to differential gene expression, including enrichment in Wnt signaling. Furthermore, mice with this syndrome exhibit behavioral inflexibility and social deficits during the postnatal period [437, 438]. Also, the knockdown of *EHMT1* in NSCs promotes a differentiated state [439]. A novel inbred strain BTBR/R idiopathic ASD model gains copy number variants (CNVs) in HDAC1, contributing to epigenetic reprogramming and immune dysfunction during embryogenesis at E11.5 [440, 441]. In addition, impairments in HDAC are involved in *SHANK3* mutations in Phelan-McDermid syndrome [442]. Prenatal HDAC inhibition by VPA transiently reduces the expression of *Shank3* 14 days after culturing embryonic neurons [263], which suggests the presence of a compensatory mechanism that reduces the genetic load for ASD, in contrast to the strong impact of * Shank3* mutations in Phelan-McDermid syndrome. Interestingly, * Shank3* expression and protein levels are lower during postnatal development of the cerebellum in mice exposed to prenatal VPA [367]. This indicates that differences could be established in specific structures in the postnatal brain. Moreover, *MECP2* mutations in Rett syndrome and ASD models highlight the importance of genetic variants that result in impairments in dependence on the epigenetic background. MecP2 binds to methyl CpG sites in the promoter region of several genes, which mainly leads to gene repression but can also result in gene expression depending on its interaction with co-factors [435]. In contrast, prenatal exposure to VPA at E12.5, but not E14.5, downregulated the expression of *Mecp2* and increased the expression of *Bdnf* and miR132 after exposure but not at birth [368]. Interestingly, this study suggests that MecP2 expression would not be directly regulated by HDACi. Instead, it was reported that HDACi increased *Bdnf* expression, increasing miR132 expression through a downstream transcription factor. As previously reported, increased miR132 expression leads to the downregulation of *Mecp2* expression [443]. This explains the immediate BDNF expression one hour after VPA until six hours, but only mir-132 shows sustained expression 24 hours later [368]. Moreover, the MecP2-BDNF-miR132 regulatory feedback loop was found to be altered in postmortem brain tissue from individuals with Rett syndrome [444]. In addition, exposure to VPA increased CpG promoter demethylation of *Reln* and *Gad67*, as well as H3ac binding. This could be attributed to the activity of HDACi and demethylation [384, 385, 392], which contribute to the epigenetic load caused by VPA exposure. Overall, these monogenic and environmental ASD models recapitulate several points of convergence with prenatal exposure to VPA and the interplay of genetic-epigenetic interactions during brain development.

As summarized in Table **2**, prenatal exposure to the VPA model has been shown to explain certain aspects of ASD etiology, especially the regulation of epigenomics through histone acetylation. This model also involves downstream pathways, such as Wnt signaling, during gestation, which affect various brain developmental processes, including the proliferation of NPCs, differentiation, and cellular organization in the cerebral cortex. Some of these impairments in differentiation could be triggered after exposure to VPA, leading to a reprogramming of transcriptional profiles. This reprogramming may result in increased glutamatergic differentiation and impairments in GABAergic and oligodendroglial lineages. Epigenetic modifications, such as histone acetylation, induce immediate and transient differential gene expression, while DNA and histone methylation could later sustain it. Also, it has been suggested that VPA, through Wnt signaling, could regulate the expression of redox proteins such as Trx1/2, leading to an increase in oxidative stress. Overall, these early events during gestational development could later result in an imbalance between E/I signals as well as impaired immune function and connectivity. Still, it is unclear how these early gestational events selectively impact certain structures that contribute to the core ASD symptoms in the postnatal brain. However, both gestational and postnatal impairments reported by prenatal exposure to VPA align with common pathological processes observed in ASD subjects. Interestingly, other environmental and monogenic-induced models of ASD converge on several mechanisms that could explain a common physiopathology [10, 202, 264, 409, 445].

## 8 PERSPECTIVES AND LIMITATIONS

The prenatal VPA exposure model has consistently recapitulated core ASD symptoms and neurobiological impairments that have been reported in multiple studies involving human and animal models. The significant variability reported in the model should be carefully considered to establish more reproducible results. The *in vivo* studies would consider the period of embryonic development, which may affect the outcome after VPA administration. This includes the effect on neural progenitor differentiation processes and the reported behavioral disturbances. Some of these changes appear to be temporary, so the timing of the evaluation should also be taken into consideration. In summary of the articles discussed here, a dose of 400-600 mg/kg and embryonic exposure time between E11.5 and E12.5 seem to be reasonable thresholds, as previously suggested [80]. Interestingly, when considering this VPA threshold, consistent impairments in two core behaviors of ASD were reported across postnatal developmental stages. Given the limited number of studies reporting autism-like behaviors resulting from prolonged prenatal or early postnatal exposure, these models should not be discarded. Instead, they should be further classified as single or sustained prenatal and postnatal exposures to VPA, and the neurobiological correlates should be carefully compared. In general, for VPA and other ASD models, behavioral evidence can be enhanced by standardizing current protocols and expanding specific domains. These domains include semi-natural habitat social behavior, decision-making, cognitive flexibility, perseverative behavior, and sensory processing and integration [80, 446-448]. Furthermore, animal models of ASD should increase their focus on endophenotypes, which are characteristics that may have a genetic relationship to a disorder without necessarily predicting a diagnosis. Identifying the points of convergence and divergence between monogenic and environmental models will help us better understand the complex and heterogeneous etiology of ASD. For example, seizure susceptibility, anxiety-like behaviors, abnormalities in sensory processing, and sleep disturbances are frequently reported endophenotypes in ASD animal models. Prenatal exposure to VPA exposure is a common factor that converges with monogenic and other environmental models [449, 450].

The *in vitro* studies would consider the standardized dose and time of administration of VPA, as well as the days after exposure. This is because concentration and time dependence in transcriptional profiles by HDACi, including VPA, have been previously reported [451]. Interestingly, this study demonstrated an adaptive response to epigenomic disruption by HDACi, including the downregulation of lysine acetyltransferases (KATs) in a dose-dependent manner. Considering that most *in vitro* studies have been conducted using cells of embryonic origin, iPSCs, or NPCs to assess the level of neuronal differentiation, it seems that the dosage is the least significant variation factor. Commonly used concentrations include 0.2, 0.5, 1, and 2 mM, with similar results observed in the 0.5-1 mM range. On the other hand, the duration of exposure and the number of days after exposure have been identified as the primary sources of variation across different studies, with both ranging from hours to days. Cell genomic programs during pluripotency and the proliferative state are different compared to differentiated cells, which are compromised by VPA HDACi and should be taken into consideration. Many of the *in vitro* studies considered in this review focus on the effect of VPA on neural development and ASD. These studies primarily use proliferative cells, such as embryonic progenitors or iPCs, that have been reprogrammed to exhibit a neural phenotype. To mechanistically explain the epigenetic regulation of VPA, several studies were conducted on cell lines, including tumor-derived cell lines with proliferative capacity.

It is important to note that the VPA model has limitations, and it is unclear if the variation between the VPA models represents a wide spectrum of ASD or more technical differences in approach among different laboratories. The specific causes of autism are still largely unknown, and it is reasonable to assume that most patients were not exposed to VPA during gestation. This raises questions about the validity of the VPA model in relation to human autism. On the other hand, highly penetrant ASD-related gene variants have not been identified in the majority of cases, accounting for only about 5-15% of ASD cases [270, 452]. Also, some of these gene variants are related to specific clinical features unrelated to ASD. Interestingly, these monogenic disorders share comorbidities with idiopathic ASD cases, which may be attributed to epigenomic reprogramming and impairments during embryonic development, both of which are associated with exposure to VPA.

## CONCLUSION

The prenatal exposure to the VPA model has been a valuable tool for studying the etiology and physiopathology related to ASD over the past three decades. This model has garnered research support, demonstrating both construct validity and face validity as an animal model. Since the first studies suggested multiple targets and interactions of VPA, there has been a better understanding of a complex series of events during embryonic development. These events begin with epigenetic modifications and later involve the reprogramming of transcriptional profiles following HDAC inhibition. This conceptualizes the framework in which this model fits into the epigenetic-genetic interplay in brain development. It contributes to the understanding of the etiology of ASD and subsequent disturbances in the postnatal brain, including neuronal organization and architecture, immune dysregulation, and imbalances in the functioning of excitatory/inhibitory systems. These disturbances are mainly caused by synapse signaling and interact with other environmental and genetic models. Thus, combining human and animal studies has helped us better understand the molecular and neurobiological mechanisms associated with the behavioral phenotypes of ASD.

## Figures and Tables

**Table 1:** Autism-like behaviors by prenatal VPA exposure model across different species.

-			**Behavioral Trait Evaluated**	
**VPA Dose and ** **Time Exposure**	**Specie**	**Developmental Stage Tested**	**Cluster A: Social & Communication Deficits (References)**	**Cluster B: Restrictive and Perseverative Behaviors (References)**
Between 400-600 mg/kg Around E11.5-12.5	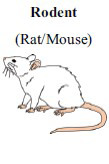	Newborn-Infant Rat/Mouse P0 > P19	● Ultrasonic vocalizations [83-92] ● Free social interaction [93]	● Open-field repetitive behaviors [93]
		Juvenile Rat P20 >P39 Mouse P20 > P29	● Social preference [73, 85, 87, 89, 94-120] ● Social Novelty [73, 85, 87, 89, 90, 94-113, 115-123] ● Free social interaction [84, 86, 87, 89, 90, 93, 101, 106, 111, 114, 116, 120, 124-137] 22/10/23 3:49 PM	● Open-field repetitive behaviors [87, 89, 94, 95, 99, 103, 106-112, 116, 117, 119, 122-125, 127, 128, 130, 131, 133, 134, 137-141] ● Marble Burying [104, 116, 142] ● Inflexibility by T or Y maze [84, 101, 111, 129, 132]
		Pubertal Rat P40 > P69 Mouse P30 > P59	● Social preference [83, 85, 95, 97, 98, 102, 104, 104, 105, 107, 108, 110, 111, 113, 115-117, 119-122, 143-159] ● Social Novelty [85, 95, 97, 98, 102, 104, 107, 110, 111, 113, 117, 119-122, 145, 150-153, 155-160] ● Free social interaction [86, 111, 116, 120, 124, 125, 128-130, 134, 135, 137, 161-170]	● Open-field repetitive behaviors [85, 95, 107, 108, 111, 116, 117, 119, 122, 124, 125, 128, 130, 134, 137, 140, 143, 146, 147, 150-152, 152-155, 157-161, 169-174] ● Marble Burying [104, 116, 148, 158, 170] ● Inflexibility by T or Y maze [111, 129, 145, 150-152, 155, 156, 159, 163, 168]
		Adult Rat P70 > Mouse P60 >	● Social preference [83, 89, 101, 104, 114, 115, 120, 145, 149, 154, 175-179] ● Social Novelty [89, 104, 120, 145, 154, 175, 179] ● Free social interaction [89, 90, 101, 114, 120, 124, 134, 135, 167, 180, 181]	● Open-field repetitive behaviors [89, 124, 134, 138, 140, 154, 180-183] ● Marble Burying [104] ● Inflexibility by T or Y maze [101, 104, 145]
Between 200-300 mg/kg Daily E60-68 [184] Double: E26 & 29 [185] Daily E60-66 [186]	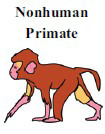	Infant 3 months^+^	● Vocalizations [184]	-
		Juvenile 17-21 months^++^	● Social visual preference [185] ● Free social interaction [185]	● Stereotypic circling behavior [185]
		Adult 1.4-2.2 years^+^	● Third-party social reciprocity [186]	-
35 µmol p/egg Embryo egg day 14	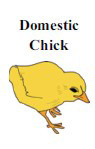	Newborn to 1^st^ week of age	● Social preference [187, 188] ● Social visual preference [189] ● Social attachment [190] ● Free social interaction [191] ● Vocalizations [191, 192]	-
		2^nd^ to 3^rd^ week of age	● Social (familiar) preference [192]	-
Between 5-75 µM Long exposure 8-108 hpf [193] 4-120 hpf [194] 0-48 hpf [195] 10-24 hpf [196]	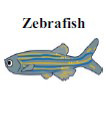	Young 5-21 dpf	● Social preference [194] ● Social visual preference [193] ● Free social interaction [193] ● Shoaling behavior [193]	● Stereotypic circling behavior [194]
		Adult 70 & 120 dpf [195] 6 mpf [196]	● Social preference [195, 196]	-

**Note:** Studies conducted are classified according to three criteria: **1**) Group of species tested, such as rodents, including mice and rats from different strains; **2**) Developmental stage when the behavioral test was conducted for each species. Rodent developmental stages were considered based on sexual development and typical gonadal hormone-sensitive social behavior in comparison to humans [197]. Studies involving nonhuman primates, domestic chicks, and zebrafish examined the stage of development relevant to each study; **3**) Autism-like behaviors were grouped and categorized based on the two diagnostic criteria outlined in DSM-V-TR [66]. Variations on behavioral tests were grouped based on the common core of impairments reported. From **Cluster A: ** Social preference: The preference to choose social stimuli, usually an animal from the same species, over neutral or nonsocial stimuli like an empty space or object. Social novelty: The preference to choose novel social stimuli over familiar ones. In domestic chicks, social familiarity is expected, which leads to opposing behavior. Free social interaction: This behavioral test measures typical social behaviors in each species, allowing animals to interact freely. Vocalizations: An analysis of the pattern and frequency of vocal calls to communicate with each other, or with its mother. Social visual preference: The tendency to choose an image or object with typical social characteristics of each species, such as faces or shapes. Third-party social reciprocity: Individuals discriminated between human actors who reciprocated in social exchanges and those who did not. Social attachment: The adaptive or learned formation of social bonds with specific individuals. Shoaling behavior: The tendency of fish to form groups or schools and swim together in a coordinated manner. From **Cluster B: **Open-field repetitive behavior: Frequency of behaviors, typically in an open field [198], which allows measuring behaviors outside of a familiar home cage arena and includes repetitive movements, rearing, self-grooming, locomotion, or hyperactivity. Marble burying: Frequency of behaviors exhibited in a familiar home cage environment (with bedding material) and unfamiliar objects that elicit digging behavior toward the novel object. Common behaviors reported: The number of marbles buried, time and frequency of burying, digging, or self-grooming. Inflexibility in the T or Y maze: The test requires the animals to switch to a new reward location between trials, thus assessing their behavioral flexibility. Stereotypic circling behavior: Repetitive and circular movement patterns exhibited by animals, including primates, mice, and zebrafish. *Terminology:* E, embryo, or gestational day (E11.5); P, postnatal day (P3); hpf, hours post-fertilization; dpf, days post-fertilization; mfp, months post-fertilization. ^+^Marmoset monkey (*Callithrix jacchus*), ^++^Cynomologus monkey (*Macaca fascicularis*).

**Table 2:** Neurobiological and molecular impairments caused by VPA exposure through *in vivo* and *in vitro* studies.

**Category**	**Rodent**	**Nonhuman ** **Primate**	**Zebrafish**	***In Vitro* Studies (2D Culture & Brain Organoids)**
Histone acetylation/methylation and Chromatin remodeling	● ↑ H3Kac and H4Kac [122, 130, 361] ● ↑ H3Kme and H4Kme [362] ● ↓ H3Kme and H4Kme [362] ● Chromatin remodeling [363]	-	● ↑ H3Kac and H4Kac [233, 364]	● ↑ H3Kac and H4Kac [365] ● ↑ H3Kme and H4Kme [366] ● Chromatin remodeling [365]
ASD-associated genes expression	● *Chd7* [313], *Shank2*, *Shank3*, *Nlgn3* [336, 367], *Mecp2* [368]	● *SHANK3*, *SHANK1* [185]	● *shank3*, *nrxn1*, *nlgn3* [194]	● *Shank2-3*, *Nlgn1* [263], *Cntnap2* [244, 263], *FOXP1*, *RELN*, *CHD7*, *CHD8*, *NLGN2-3*, *TSC1-2, * *SHANK1-3* [365]
Neurogenesis and cell density	● ↑ Proliferation [122, 222] ● ↑ Cortical cell density [88] ● ↓ Proliferation [130, 230] ● ↓ Cortical cell density [88, 130, 230] ● ↑ Neuronal phenotype differentiation [122, 222]	● ↓ Proliferation [185] ● ↓ Cortical cell density [185] 22/10/23 3:49 PM	● ↑ Proliferation [262] ● ↓ Proliferation [233] ● ↑Neuronal phenotype differentiation [194] ● ↓Neuronal phenotype differentiation [232, 262] 22/10/23 3:49 PM	● ↑ Proliferation [222] ● ↓ Proliferation [12, 221, 234, 369] ● ↓ Cortical cell density [11, 12, 234] ● ↑ Neuronal phenotype differentiation [221, 234, 369]
Excitatory/ Inhibitory Imbalance	● ↑ Glutamatergic neuronal excitability [131, 284, 285, 288, 289] ● ↓ Glutamatergic neuronal excitability [283, 286, 291] ● ↑ Glutamatergic neuronal density [122] ● Altered synaptic protein expression [95, 122, 284, 291, 336, 337, 361] ● ↓ GABAergic neuronal density [229]	● Altered synaptic protein expression [185]	-	● ↑ Glutamatergic neuronal density [234] ● Altered synaptic protein expression [11, 361]
Oligodendroglia and Myelination impairments	● ↓ OL density or associated gene/protein expression [83, 299, 311-313] ● ↓ Myelin density or gene/protein expression [83, 299, 311, 312]	-	-	-
Immune and Oxidative stress impairments	● ↑ Oxidative stress [336] ● ↑ Microglia density [149, 154, 311, 335] ● ↓ Microglia density [338] ● ↓ Pro-inflammatory cytokines [154, 336]	-	-	-

**Note:** Studies conducted *in vivo* considered prenatal VPA exposure in a single dose during embryonic development (rodent and non-human primate) and the first five days postfertilization (zebrafish), reporting impairments during the gestational or postnatal period. Studies conducted *in vitro* considered VPA exposure over embryonic progenitor cells (EPCs), neural progenitor cells (NPCs), or induced pluripotent stem cells (iPSCs) under neural differentiation in 2D cultures and brain organoids. ASD-associated genes were considered according to the top ranking in the Autism Informatics Portal (AutDB) [370].

## LIST OF ABBREVIATIONS

ASD: Autism Spectrum Disorder
BBB: Blood-brain Barrier
BTBR/R: Inbred Mouse Strain BTBR TF/ArtRbrc
cKO: Conditional Knock-out
CNV: Copy Number Variant
CpG: Cytosine Guanine Dinucleotide
DMNT1: DNA Methyltransferase 1
dpf: Days Post-fertilization
E,DSM-V-TR: Diagnostic Statistics and Mental Health Disorders V-Text Revised Embryonal day
E/I: Excitatory/Inhibitory Imbalance
EHMT1: Histone Methyltransferase
FVS: Fetal Valproate Syndrome
GSK-3β: Glycogen Synthase Kinase-3β
H3/H4Kac: Histone Lysine Acetylation
H4/H4Kme: Histone Lysine Methylation
HDACi: Histone Deacetylase Inhibition
HDM: Histone Demethylase
hiPSC: Human Induced Pluripotent Stem Cell
HPF: Hours Post-fertilization
MFP: Months Post-fertilization
MIA: Maternal Immune Activation
mRNA: Messenger RNA
NO: Nitric Oxide
NPC: Neural Progenitor Cells
NSC: Neural Stem Cells
P: Postnatal Day
PFC: Prefrontal Cortex
ROS: Reactive Oxygen Species
TSA: Trichostatin A
VPA: Valproate
VPD: Valpromide
